# The Connected Intensive Care Unit Patient: Exploratory Analyses and Cohort Discovery From a Critical Care Telemedicine Database

**DOI:** 10.2196/13006

**Published:** 2019-01-24

**Authors:** Patrick Essay, Tala B Shahin, Baran Balkan, Jarrod Mosier, Vignesh Subbian

**Affiliations:** 1 College of Engineering The University of Arizona Tucson, AZ United States; 2 College of Medicine - Tucson The University of Arizona Tucson, AZ United States; 3 Division of Pulmonary, Allergy, Critical Care, and Sleep Department of Medicine The University of Arizona Tucson, AZ United States; 4 Department of Emergency Medicine The University of Arizona Tucson, AZ United States; 5 Department of Systems and Industrial Engineering The University of Arizona Tucson, AZ United States; 6 Department of Biomedical Engineering The University of Arizona Tucson, AZ United States

**Keywords:** telemedicine, critical care, medical informatics applications, intensive care units

## Abstract

**Background:**

Many intensive care units (ICUs) utilize telemedicine in response to an expanding critical care patient population, off-hours coverage, and intensivist shortages, particularly in rural facilities. Advances in digital health technologies, among other reasons, have led to the integration of active, well-networked critical care telemedicine (tele-ICU) systems across the United States, which in turn, provide the ability to generate large-scale remote monitoring data from critically ill patients.

**Objective:**

The objective of this study was to explore opportunities and challenges of utilizing multisite, multimodal data acquired through critical care telemedicine. Using a publicly available tele-ICU, or electronic ICU (eICU), database, we illustrated the quality and potential uses of remote monitoring data, including cohort discovery for secondary research.

**Methods:**

Exploratory analyses were performed on the eICU Collaborative Research Database that includes deidentified clinical data collected from adult patients admitted to ICUs between 2014 and 2015. Patient and ICU characteristics, top admission diagnoses, and predictions from clinical scoring systems were extracted and analyzed. Additionally, a case study on respiratory failure patients was conducted to demonstrate research prospects using tele-ICU data.

**Results:**

The eICU database spans more than 200 hospitals and over 139,000 ICU patients across the United States with wide-ranging clinical data and diagnoses. Although mixed medical-surgical ICU was the most common critical care setting, patients with cardiovascular conditions accounted for more than 20% of ICU stays, and those with neurological or respiratory illness accounted for nearly 15% of ICU unit stays. The case study on respiratory failure patients showed that cohort discovery using the eICU database can be highly specific, albeit potentially limiting in terms of data provenance and sparsity for certain types of clinical questions.

**Conclusions:**

Large-scale remote monitoring data sources, such as the eICU database, have a strong potential to advance the role of critical care telemedicine by serving as a testbed for secondary research as well as for developing and testing tools, including predictive and prescriptive analytical solutions and decision support systems. The resulting tools will also inform coordination of care for critically ill patients, intensivist coverage, and the overall process of critical care telemedicine.

## Introduction

Critical care telemedicine, or tele-ICU, is broadly defined as a collaborative, interprofessional care model for critically ill patients where the bedside intensive care unit (ICU) team and patient are networked to a centralized and often remotely located critical care team using telecommunication and computer systems [1,2]. Applications of tele-ICU include quality improvement, continuous monitoring of patients for early warning of deterioration, and varying degrees of clinical decision support, interventions, and consultations [3,4]. Although there exist several tele-ICU models [5,6], we refer to tele-ICU in the context of continuous patient monitoring and subsequent data generation from application of telemedicine in intensive care settings as opposed to more active models involving computer-generated alerts or those with interventions such as audio and video consultations.

Advances in data management infrastructure, biomedical sensors and devices, and computational methods, coupled with the current trend of consolidation of hospitals into large health care delivery systems, provide unique opportunities for not only enhancing tele-ICU capabilities to improve patient, physician, and system-level outcomes but also leveraging tele-ICU data for research and evaluation purposes. The full benefit of the influx of tele-ICU data, however, has yet to be realized.

The objective of this study was to explore opportunities and challenges of using multisite, multimodal data acquired through critical care telemedicine. Using a publicly available tele-ICU database (eICU Collaborative Research Database), we illustrate the quality and potential uses of remote monitoring data [7]. In addition, we present a case study on extraction of multiple respiratory failure patient cohorts to illustrate various strengths and limitations of the database. Specifically, we present 3 patient cohorts—endotracheal intubation patients, patients requiring other noninvasive ventilation therapy, and patients with both invasive and noninvasive treatments in the same visit—and attempt to generate relevant questions for further research.

## Methods

The electronic ICU (eICU) database consists of deidentified data collected from patients admitted to adult ICUs between 2014 and 2015. It consists of a wide array of data from admission diagnosis, patient severity scores, standard and custom lab values, nurse charting, physiological data, and treatment records through discharge status. Clinical scores in the database include the Acute Physiology Score (APS) III and the Acute Physiology and Chronic Health Evaluation (APACHE) IV and IVa, both of which are examples of existing instruments that have been widely used in critical care settings for assessment of disease severity and outcome prediction [8].

Hospital data were extracted along with patient demographics, diagnoses, length of stay and mortality outcomes, and treatment records. APACHE IVa severity scores and prediction values were also extracted. Development of respiratory failure cohorts utilized multiple record types in the database that contain respiratory chart and treatment data. The specific cohorts were created using intubation and ventilator-type records. We then attempted to verify patients that required endotracheal intubation or noninvasive respiratory therapy with a redundant record within the database to validate that patients actually required ventilation. For example, one can confidently say that a patient with a record of *endotracheal tube* and a treatment record of *endotracheal tube insertion* was in fact intubated during their ICU stay compared with a patient who has ventilator setting records but no other indication of airway type or noninvasive respiratory therapy.

Data from the eICU database were extracted and preprocessed in Python version 2.7.14 using the Pandas [9] and Seaborn libraries [10], versions 0.23.4 and 0.9.0, respectively. A complete evaluation of all data tables in the eICU database is available [11].

## Results

### Participant Characteristics

**Figure 1:**
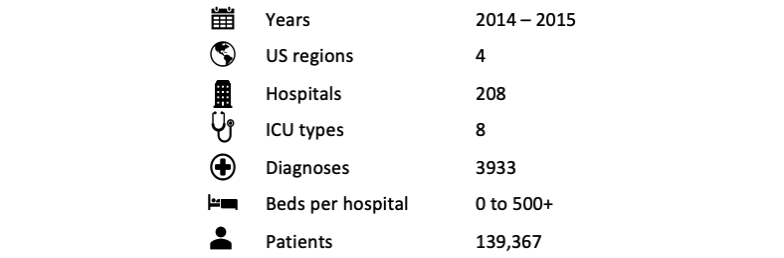
Infographic overview of the eICU Collaborative Research Database. ICU: intensive care unit.

The eICU database consists of 200,859 adult ICU stays at 208 hospitals including 139,367 unique patients with nearly equal numbers of male and female patients. The majority of patients are white. A high-level overview of the database is shown in Figure 1 and additional patient characteristics are presented in Table 1.

**Table 1:** Basic patient characteristics in the critical care telemedicine (tele-ICU) database.

Variable		Level	Overall
Unique patients, n		—^a^	139,367
Distinct ICU^b^ admissions, n		—	200,859
Age, years, mean (SD)		—	62.1 (16.7)
**Gender, n (%)**			
	Male	—	108,379 (53.96)
	Female	—	92,303 (45.95)
	Other	—	134 (00.09)
**Race, n (%)**			
	White	—	155,285 (77.31)
	African American	—	21,308 (10.61)
	Hispanic	—	7464 (3.72)
	Asian	—	3270 (1.63)
	Native American	—	1700 (0.85)
	Unknown or unspecified	—	11,832 (5.90)
ICU length of stay in days, mean (IQR^c^)		ICU	3.00 (2.31)
ICU mortality, % of ICU admissions		ICU	5.79
Hospital length of stay in days, mean (IQR)		Hospital	8.06 (7.04)
Hospital mortality, % of admissions		Hospital	9.24

^a^Not applicable.

^b^ICU: intensive care unit.

^c^IQR: interquartile range.

**Figure 2:**
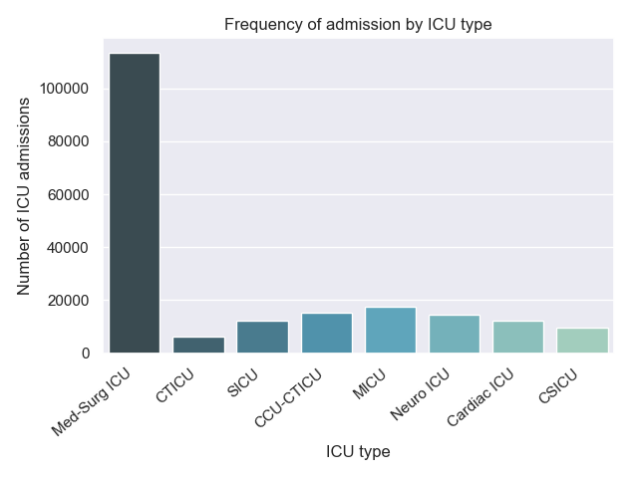
Frequency of admission to each intensive care unit type within the eICU Collaborative Research Database. ICU: intensive care unit; Med-Surg ICU: medical surgical ICU; CTICU: cardiothoracic ICU; SICU: surgical ICU; CCU-CTICU: coronary care/CTICU ICU; MICU: medical ICU; Neuro ICU: neurological ICU; Cardiac ICU: cardiological ICU; CSICU: cardiac surgery ICU.

**Figure 3:**
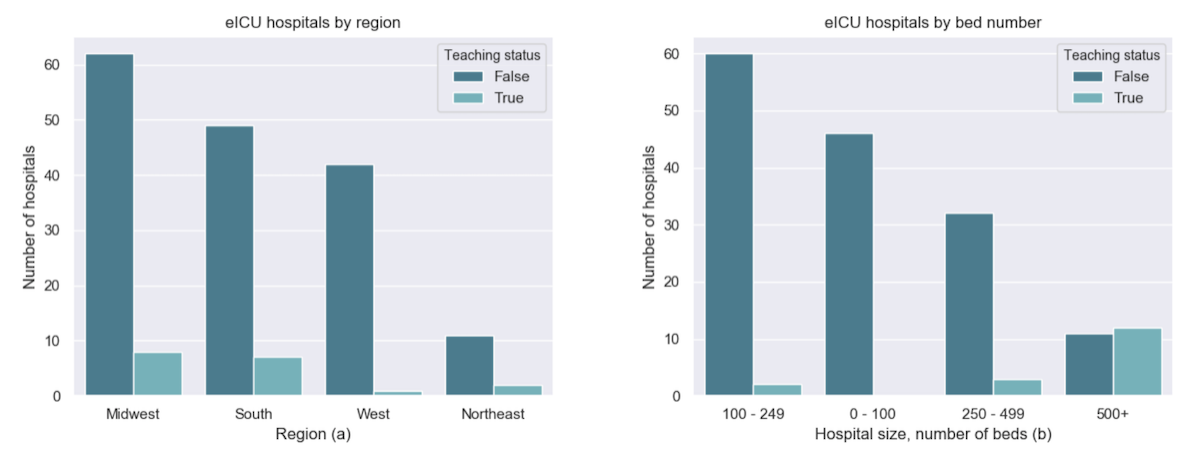
(a) Hospital distribution by size and associated teaching status (b) hospital distribution by United States region and associated teaching status.

The ICU types covered in the database are wide ranging, with mixed medical-surgical ICU as the most common critical care setting (Figure 2). This is likely because of the configuration and workflow of ICUs within each hospital. The majority of hospitals in the eICU database are primarily nonteaching hospitals across most of the United States (Figure 3).

There were 431 admission diagnoses with several additional diagnosis records in the database that provide context and higher granularity to the reasons for admission. Patients with cardiovascular conditions accounted for more than 20% of ICU stays, and those with neurological or respiratory illness accounted for nearly 15% of ICU unit stays. Table 2 shows further details on the most frequent admission diagnosis by number of ICU stays and the associated percent of the total visits in the database with corresponding mortality rates and average ICU length of stay.

**Table 2:** Most frequent admission diagnosis categories with corresponding intensive care unit (ICU) mortality rate and average ICU length of stay.

Admission diagnosis name	ICU stays, n (%)	Average length of stay, days	Mortality, n (%)
Cardiovascular	79,560 (20.6)	2.97	4861 (7.33)
Neurologic	31,113 (8.07)	2.83	949 (3.64)
Respiratory	25,813 (6.69)	3.68	1408 (7.00)
Gastrointestinal	17,726 (4.60)	2.95	681 (4.63)
Sepsis, pulmonary	8862 (2.30)	4.31	904 (12.26)
Metabolic or endocrine	8025 (2.08)	1.88	72 (1.06)
Infarction, acute myocardial	7228 (1.87)	2.09	180 (2.93)
Trauma	7136 (1.85)	3.59	303 (5.01)
Cerebrovascular accident or stroke	6647 (1.72)	2.77	290 (5.20)
Congestive heart failure	6617 (1.72)	3.13	302 (5.67)

**Table 3:** Overview of Acute Physiology Score and Acute Physiology and Chronic Health Evaluation (APACHE) scores in the tele-ICU (critical care telemedicine) database with APACHE IVa predictions.

Variable	Overall	Predicted^a^	Actual
Acute Physiology Score, mean (IQR^b^)	43.63 (27.00)	—^c^	—
APACHE Score, mean (IQR)	55.49 (31.00)	—	—
Intensive care unit (ICU) length of stay in days, mean (IQR)	—	3.87 (3.02)	3.00 (2.31)
ICU mortality, % of ICU admissions^d^	—	5.49	5.79
Hospital length of stay in days, mean (IQR)	—	9.44 (5.88)	8.06 (7.04)
Hospital mortality, % of admissions^d^	—	3.84	9.24

^a^Prediction values taken from APACHE version IVa.

^b^IQR: interquartile range.

^c^N/A: not applicable.

^d^Predicted ICU and hospital mortality values are the averages of percent chance of dying of all patients.

### Severity and Predictive Scoring Systems

**Figure 4:**
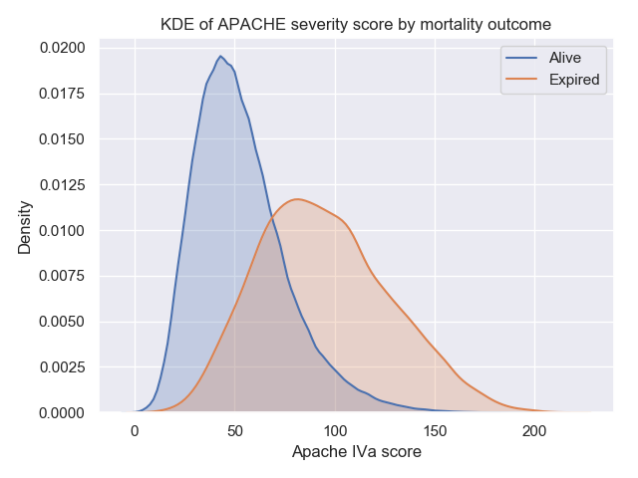
Kernel density estimate (KDE) of Acute Physiology and Chronic Health Evaluation (APACHE) IVa scores within the eICU Collaborative Research Database stratified by actual intensive care unit mortality outcome.

Severity of illness and prognosis are captured in the eICU database as a function of the APACHE IVa score and consists of 288,090 entries. The APACHE evaluation also provides predictions of patient outcomes soon after ICU admission and includes probability of mortality, length of stay, and ventilation days and is used in conjunction with APS. An overview of APS and APACHE scores is presented in Table 3. The distributions of patient severity within the eICU database as a function of APACHE IVa stratified by discharge status of alive or expired is shown in Figure 4.

**Figure 5:**
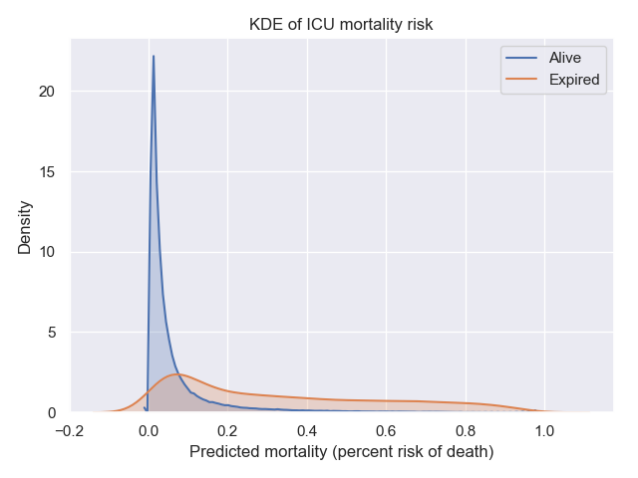
Kernel density estimate (KDE) of predicted hospital mortality in the eICU Collaborative Research Database stratified by actual intensive care unit (ICU) mortality outcome.

The APACHE mortality prediction distributions were normalized and segregated by discharge status as shown in Figure 5. This illustrates existing model deficiencies where predicted mortality is not reliable at higher severities [12,13]. Although the predictions for the survivors are reasonably accurate, the predictions for nonsurvivors are not. We include this to illustrate that although predictive models are useful in certain situations, they may not perform well in others because of the dynamics involved or issues with source data [14]. These results are consistent with evaluations of earlier versions of APACHE predictions [15] and are an area of improvement for tele-ICU to provide the best possible decision support for the fast-paced ICU environment.

### Case Study on Respiratory Failure Patients

**Figure 6:**
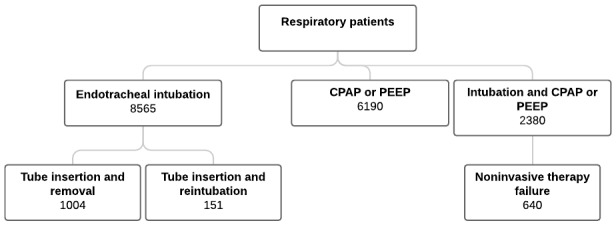
Number of patients with particular respiratory-type treatment records in the eICU database. CPAP: continuous positive airway pressure; PEEP: positive end expiratory pressure.

The selected respiratory failure patient cohorts and corresponding number of patients within each group developed from treatment records are shown in Figure 6. Possible noninvasive ventilation therapy failure was determined using treatment timestamps. Many endotracheal intubation records correspond to continuous positive airway pressure (CPAP) and positive end expiratory pressure (PEEP) records at the same time. However, it is possible that patients with intubation treatment recorded after CPAP or PEEP treatment required intubation after failure of noninvasive respiratory therapy.

**Figure 7:**
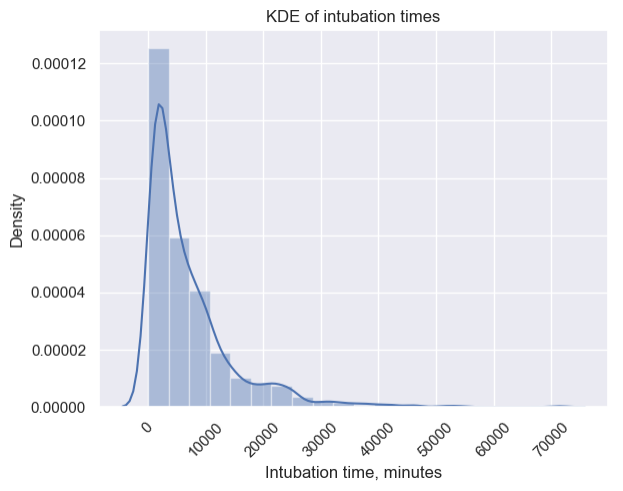
Kernel density estimate (KDE) of intubation times for patients with endotracheal tube insertion and removal.

**Figure 8:**
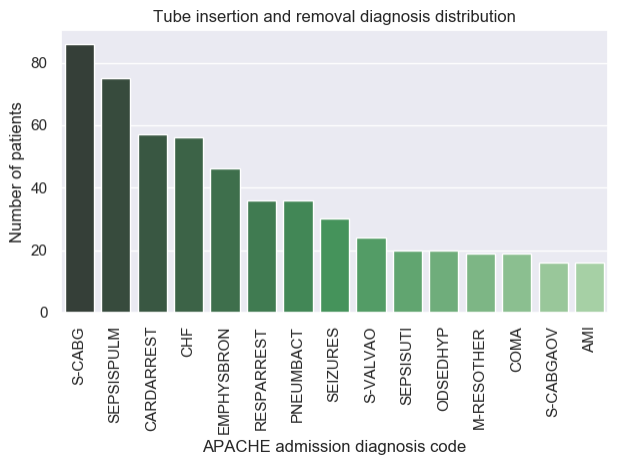
Fifteen most frequent admission diagnoses for patients with record of endotracheal tube insertion and tube removal. AMI: acute myocardial infarction; CABG: coronary artery bypass grafting; CHF: congestive heart failure.

As a demonstration of database coverage and specificity within a particular patient cohort, we selected the 1004 patients that have definitive records of both endotracheal tube insertion and removal. Using the associated treatment time stamps for tube insertion and removal, intubation times were estimated as was the distribution of admission diagnoses across the same cohort (Figures 7 and 8).

## Discussion

### Principal Findings

Investigation of the eICU Collaborative Research Database shows a wide range of illnesses from a large number of hospitals that span the continental United States. Organized as a relational database, it is highly versatile for narrowing research focus to specific critical care patient populations, and it allows for robust and generalizable analysis and modeling across multiple institutions and regions. The case study on respiratory failure patients illustrates the potential for cohort discovery and analysis of specific patient subgroups (see Figure 6) using unique identifiers across the database, coupled with the ability to query multiple record types such as treatment records, respiratory, medication, or laboratory data. For example, if using *treatment* records, one would find 8565 unique patients that required endotracheal intubation. If searching for patients with distinct records of both endotracheal tube insertion and removal treatments, the available cohort is limited to 1004 patients. Any combination of these data with other record types may limit or extend cohort size further.

Although the eICU database provides real-word critical care data from a diverse sample of hospitals and practice settings to evaluate interventions, there are some limitations to consider. First, the granularity of the data can be limiting, given the nature of data collection. For example, despite continuous collection of hemodynamic data, interventions such as tracheal intubation may be recorded with a margin of error because of a requirement for manual entry of events into the electronic medical record. Narrowing the window between when the intubation was performed and when the event was recorded could potentially be accomplished by using drugs associated with intubation. Regardless, this limitation makes studying peri-intubation complications difficult as one does not know whether a hemodynamic decompensation occurred before or after the intubation procedure. In addition, manually entered data could have deviations based on hospital-specific practices and protocol variations.

Second, though the eICU database is considered tele-ICU data, the mode of data collection and the origins of data are not well defined. Specifically, it is not clear which data are generated at the bedside versus the remote unit and by whom. Third, terminology variations across institutions and health information systems pose an additional hurdle. A study of a previous version of eICU data showed discrepancies in standards for laboratory and microbiology data for patients with primary cardiovascular diagnosis [16]. This suggests that cohort discovery on eICU data may also need to be reconfigured based on specific research questions.

Finally, a major caveat to the eICU database is that the absence of a record does not mean an event did not occur. This is true of other similar databases; however, missing records are exacerbated in the eICU database because of data being from many different hospitals, and not all participating hospitals have interfaces in place to record all data types. Although there are methods for handling data sparsity and missing data [17,18], large quantities of missing data could negate the overall benefit of having a large number of hospitals in the database.

Despite these limitations, the most critical component of future tele-ICU operations and the eICU Collaborative Research Database is that of advanced analytics and clinical decision support. For example, cardiovascular complications arising from traumatic brain injury are common and are linked to increased morbidity and mortality [19]. Generally, monitoring the vital signs of the patient and controlling primary intracranial pathology are effective for proactive prevention of complications. Tele-ICU not only offers continuous display of vitals for remote monitoring but can also serve as a platform to (1) develop, implement, and test clinical and subclinical markers of patient decompensation and other adverse events and (2) further define the role of tele-ICU in improving the precision of electronic alerts [20]. For example, as alarm desensitization creates additional risk, much of the monitoring and resolving of alerts can be shifted to the tele-ICU [21].

Other large, publicly available databases, such as the Multiparameter Intelligent Monitoring in Intensive Care (MIMIC) database, have been widely used for secondary research purposes. MIMIC includes inpatient critical care data, spanning over 10 years from a single institution. An evaluation of the MIMIC database highlights the successful role of the database in risk assessment, medical personnel performance evaluation, and supporting development of clinical decision support systems [22]. The eICU database has strong potential for advancing the role of critical care telemedicine as MIMIC has been for bedside, inpatient critical care.

### Conclusions

This work, to our knowledge, is the first of its kind to demonstrate the potential and versatility of a publicly available, large, critical care telemedicine database. The ability to extract and analyze wide-ranging patient subgroups from remote monitoring data for secondary research is one of the key strengths of such a resource. As highlighted through the case study on respiratory patients, there are some limitations such as data provenance and sparsity, which are typical of such resources. Nonetheless, tele-ICU data are particularly useful to catalyze efforts around developing robust clinical decision support systems for critical care that can be distributed between bedside and remote care teams, as well as identifying specific patient populations and associated clinical events that would be appropriate for such *distributed* care. Secondary insults, in particular, stand to benefit from remote monitoring and advanced analytical support because they are highly time sensitive and potentially reversible in critically ill patients, if mitigated promptly. The eICU database and the resulting tools will also inform coordination of care, intensivist coverage, and the overall process of critical care telemedicine.

## Abbreviations

APACHE: Acute Physiology and Chronic Health Evaluation
APS: Acute Physiology Score
CPAP: continuous positive airway pressure
eICU: electronic intensive care unit
ICU: intensive care unit
IQR: interquartile range
MIMIC: Multiparameter Intelligent Monitoring in Intensive Care
PEEP: positive end expiratory pressure
tele-ICU: critical care telemedicine
